# Dynamic Whole-Body FDG PET/CT for Predicting Malignancy in Head and Neck Tumors and Cervical Lymphadenopathy

**DOI:** 10.3390/diagnostics15202651

**Published:** 2025-10-21

**Authors:** Gregor Horňák, André H. Dias, Ole L. Munk, Lars C. Gormsen, Jaroslav Ptáček, Pavel Karhan

**Affiliations:** 1 Department of Nuclear Medicine, University Hospital Olomouc, Palacký University Olomouc, Zdravotníků 248/7, 77900 Olomouc, Czech Republic; 2 Department of Nuclear Medicine & PET-Centre, Aarhus University Hospital, Palle-Juul-Jensens Boulevard 165, 8200 Aarhus, Denmark; 3 Department of Clinical Medicine, Aarhus University, Palle-Juul-Jensens Boulevard 99, 8200 Aarhus, Denmark; 4 Department of Medical Physics and Radiation Protection, University Hospital Olomouc, Zdravotníků 248/7, 77900 Olomouc, Czech Republic

**Keywords:** PET/CT, SUV, multiparametric PET, dynamic PET, FDG, patlak modeling, head and neck tumor, lymphadenopathy, head and neck cancer

## Abstract

**Background:** Dynamic whole-body (D-WB) FDG PET/CT is a novel technique that enables the direct reconstruction of multiparametric images representing the FDG metabolic uptake rate (MR_FDG_) and “free” FDG (DV_FDG_). Applying complementary parameters with distinct characteristics compared to static SUV images, the aims of this study are as follows: (1) to determine the threshold values of SUV, MR_FDG_, and DV_FDG_ for malignant and benign lesions; (2) to compare the specificity of MR_FDG_ and DV_FDG_ images with static SUV_bw_ images; and (3) to assess whether any of the dynamic imaging parameters correlate more significantly with malignancy or non-malignancy in the examined lesions based on the measured values obtained from D-WB FDG PET/CT. **Methods**: The study was a retrospective analysis of D-WB PET/CT data from 43 patients (23 males and 20 females) included both in the context of primary staging as well as imaging performed due to suspicion of post-therapeutic relapse or recurrence. Standard scanning was performed using a multiparametric PET acquisition protocol on a Siemens Biograph Vision 600 PET/CT scanner. Pathological findings were manually delineated, and values for SUV_bw_, MR_FDG_, and DV_FDG_ were extracted. The findings were classified and statistically evaluated based on their was histological verification of a malignant or benign lesion. Multinomial and binomial logistic regression analyses were used to find parameters for data classification in different models, employing various combinations of the input data (SUV_bw_, MR_FDG_, DV_FDG_). ROC curves were generated by changing the threshold p-value in the regression models to compare the models and determine the optimal thresholds. **Results:** Patlak PET parameters (MR_FDG_ and DV_FDG_) combined with mean SUV_bw_ achieved the highest diagnostic accuracy of 0.82 (95% CI 0.75–0.89) for malignancy detection (F1-score = 0.90). Sensitivity reached 0.85 (95% CI 0.77–0.91) and specificity 0.93 (95% CI 0.87–0.98). Classification accuracy in tumors was 0.86 (95% CI 0.78–0.92) and in lymph nodes 0.81 (95% CI 0.73–0.88). Relative contribution analysis showed that DV_FDG_ accounted for up to 65% of the classification weight. ROC analysis demonstrated AUC values above 0.8 for all models, with optimal thresholds achieving sensitivities of around 0.85 and specificities up to 0.93. Thresholds for malignancy detection were, for mean values, SUV_bw_ > 5.8 g/mL, MR_FDG_ > 0.05 µmol/mL/min, DV_FDG_ > 68%, and, for maximal values, SUV_bw_ > 8.7 g/mL, MR_FDG_ > 0.11 µmol/mL/min, DV_FDG_ > 202%. **Conclusions:** The D-WB [^18^F]FDG PET/CT images in this study highlight the potential for improved differentiation between malignant and benign lesions compared to conventional SUV_bw_ imaging in patients with locally advanced head and neck cancers presenting with cervical lymphadenopathy and carcinoma of unknown primary origin (CUP). This observation may be particularly relevant in common diagnostic dilemmas, especially in distinguishing residual or recurrent tumors from post-radiotherapy changes. Further validation in larger cohorts with histopathological confirmation is warranted, as the small sample size in this study may limit the generalizability of the findings.

## 1. Background

Positron Emission Tomography/Computed Tomography (PET/CT) hybrid imaging is one of the most widely used, modern, and rapidly developing methods primarily employed in oncological diagnostics but also for the evaluation of infection and inflammation [1].

PET/CT imaging is conventionally performed at a single time point for the most commonly used tracer, 2-deoxy-2-[^18^F]fluoro-D-glucose ([^18^F]FDG), 60 min after its administration, followed by the reconstruction of a standardized uptake value (SUV_bw_) image, assessing the semiquantitative relation of tracer uptake to glucose metabolism, normalized by the injected dose and body weight and adjusted for factors such as timing and decay correction [2,3,4].

In PET, the standardized uptake value is a widely used semiquantitative metric that reflects the concentration of the radiotracer in tissue, normalized for the administered dose and the patient’s body parameters. The most commonly reported form, SUV based on body weight (SUV_bw_), normalizes tracer uptake by the patient’s total body weight. While the abbreviation “SUV” is frequently used in clinical practice, it implicitly refers to SUV_bw_ unless otherwise specified. However, for clarity and consistency, especially in research or in comparison with alternative normalization methods such as SUV lean body mass (SUV_lbm_) or SUV body surface area (SUV_bsa_), it is important to explicitly state the normalization approach. In this study, all SUV values are calculated based on total body weight (SUV_bw_), unless otherwise indicated [5].

SUV_bw_ is influenced by a wide range of factors (such as patient body composition, time from injection, blood glucose, scanner calibration and others), impairing the overall precision, physiological reliability and repeatability of measured values [6,7].

The Patlak graphical model is a simplified form of full compartmental kinetic modeling based on the assumption of an irreversible transfer of a radiolabeled tracer from plasma into tissue. It assumes the existence of a linear phase during which a quasi-equilibrium is established between the free and specifically bound tracer fractions in tissue, while the irreversibly bound fraction accumulates over time. This approach transforms the nonlinear system of differential equations into a linear relationship between the normalized tissue concentration and the integral of the plasma input function, thus significantly simplifying quantification through linear regression. Patlak analysis is particularly well-suited for tracers with negligible efflux from tissue (e.g., [^18^F]FDG), where the primary output parameter is the influx constant (K_i_), which represents the product of membrane permeability and extraction fraction. Despite its simplifications, the Patlak model provides quantitatively robust and clinically relevant results with substantially lower computational complexity compared to full kinetic modeling [8].

To overcome the logistical and patient burdens associated with arterial blood sampling in dynamic PET imaging, population-based input functions (PBIFs) have been proposed as a practical alternative to individual arterial input functions (AIFs). PBIFs are derived from averaged time-activity curves of a representative population and scaled to individual patients using limited blood samples or image-derived input functions (IDIFs). In the context of multiparametric whole-body [^18^F]FDG PET imaging, PBIFs have demonstrated promising accuracy and reproducibility. A PBIF approach for 20 min dynamic whole-body [^18^F]FDG PET acquisition was clinically validated, showing that it enables reliable estimation of kinetic parameters such as the influx constant (K_i_) using Patlak analysis, while significantly reducing scan time and obviating the need for invasive sampling. Their results support the clinical feasibility of PBIF-based quantification in routine oncologic PET protocols, particularly when combined with optimized acquisition and reconstruction techniques [9].

Advancements in PET scanner technology and software have introduced new opportunities for PET/CT image quantification. Dynamic whole-body (D-WB) imaging is a recently developed technique for standard Field-Of-View PET/CT that involves multiple whole-body (WB) passes and the extraction of image-derived input functions (IDIF) [10,11], providing dynamic PET data for the reconstruction of WB multiparametric images based on linear Patlak analysis [8].

Multiparametric imaging supplements the standard SUV_bw_ image with two new parametric images: one displaying the effective metabolic rate of [^18^F]FDG being phosphorylated to [^18^F]FDG-6-phosphate (FDG-6-P) in the tissues (MR_FDG_), and the other displaying the distribution volume of free [^18^F]FDG in the reversible compartments and fractional blood volume (DV_FDG_) [11], unlike SUV_bw_ images, thus allowing the reader to differentiate between free and bound FDG-6-P in tissue.

Hybrid imaging with PET/CT plays a significant role in the diagnostic work-up of squamous cell carcinoma of the head and neck (HNSCC), particularly in staging challenging cases where clinical evaluation and other imaging methods can be unreliable. FDG PET/CT is also widely used to detect hidden primary tumors, assess response to chemoradiotherapy, and to detect relapsing disease [12,13].

Metastases to cervical lymph nodes from carcinoma of unknown primary origin (CUP) account for approximately 3–7% of all head and neck cancers [12,13,14,15]. Given the previously mentioned limitations of SUV_bw_-based evaluation, false-positive and false-negative results may occur, posing a significant diagnostic challenge [16,17]. False-negative results are commonly caused by factors such as the proximity of the lesion to areas with high metabolism, artifacts caused by dental prostheses, limited PET resolution, inherently low FDG avidity in some tumors, significant necrosis or cystic components of the tumor, and small lesion size [16,17]. On the other hand, inflammation and post-treatment fibrosis are the most common causes of false-positive results. The palatine tonsils are the most frequent site of both false-positive and false-negative findings on FDG-PET [17,18].

This study therefore aimed to evaluate the possible advantages of performing D-WB FDG PET multiparametric analysis of tumors in the head and neck region.

## 2. Methods

### 2.1. Patient Population

**Figure 1:**
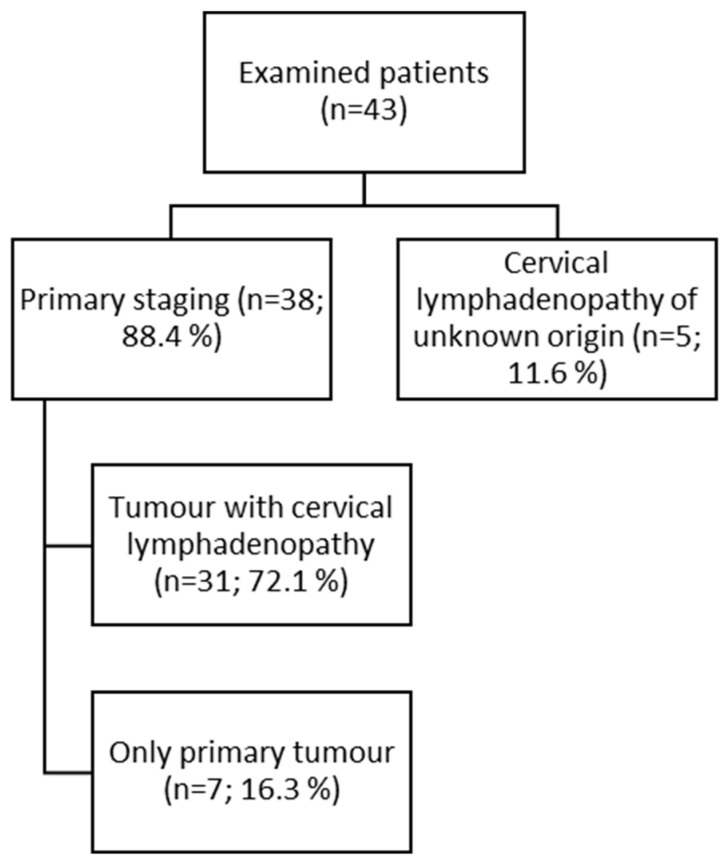
Studied group characteristics.

**Figure 2:**
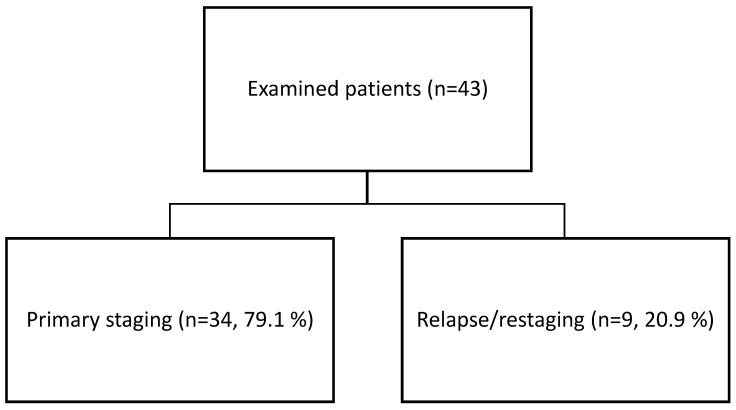
Studied group characteristics—indications.

In our dataset, patients were included both in the context of primary staging as well as imaging performed due to suspicion of post-therapeutic relapse or recurrence. The only inclusion criterion was histological verification of a malignant or benign lesion. The study was a retrospective analysis of D-WB PET/CT data from 43 patients (23 males and 20 females) selected from all D-WB PET/CT examinations performed between January 2020 and June 2021 at the Department of Nuclear Medicine & PET-Centre, Aarhus University Hospital. The majority of patients (*n* = 38; 88.4%) underwent imaging examination for tumors of the head and neck region, most of whom presented with cervical lymphadenopathy at the time of examination (*n* = 31; 72.1%). In a smaller subgroup, the indication was primary cervical lymphadenopathy of unknown origin (*n* = 5; 11.6%) (Figure 1). Figure 2 shows the distribution of indications among the patients (more details in Supplementary Table S1). Table 1 shows the histology of examined lesions.

### 2.2. Data Acquisition and Image Reconstruction

**Figure 3:**
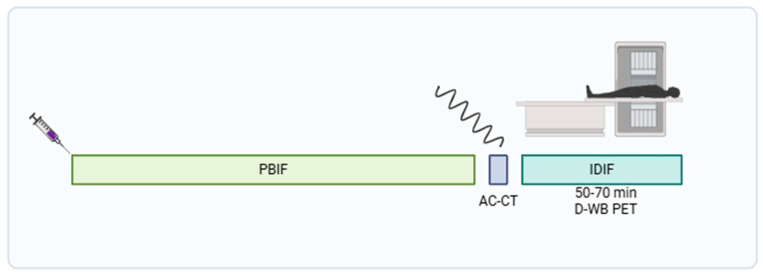
Schema of 20 min D-WB PET/CT protocol. Created with BioRender. Gregor Horňák. 2025 https://app.biorender.com/illustrations/6799f7b0a2f0a3b4ef78d1ff (accessed on 29 January 2025).

Participants were scanned using a fully automated multiparametric PET/CT acquisition protocol (FlowMotion^®^ Multiparametric PET, Siemens Healthineers, Knoxville, TN, USA) on a Siemens Biograph Vision 600 PET/CT scanner (Siemens Healthineers, Knoxville, TN, USA) with 26.2 cm axial field of view. In short, a 20 min multiparametric PET acquisition protocol using a population-based input function (PBIF) [9] scaled to the late IDIF was started 50 min after a standardized injection of FDG (4 MBq/kg) using an Intego PET Infusion System (MEDRAD, Inc., Warrendale, PA, USA). First, a low-dose WB CT (25 Ref mAs, 120 kV, Care Dose4D, Care kV, Admire level 3) was performed. The PET reconstruction parameters for D-WB were the following: For the SUV_bw_ image, we used TrueX + TOF, 6 iterations, 5 subsets, 440 × 440 matrix, no filtering, and relative scatter correction. For the dynamic PET images used for IDIF extraction, we used TrueX + TOF, 4 iterations, 5 subsets, 440 × 440 matrix, no filtering, and relative scatter correction. Parametric images of MR_FDG_ and DV_FDG_ were generated using direct Patlak reconstruction method with non-negativity constraints using list-mode data from four 5 min passes (50–70 min), TrueX + TOF, 8 iterations, 5 subsets, 30 nested loops, 440 × 440 matrix, 2 mm Gaussian filter, and relative scatter correction (Figure 3). A more detailed overview of this protocol is described by Dias et al. [9,11].

### 2.3. Image Analysis and VOI Delineation

Multiparametric images were visually inspected using Hermes Gold Client v.2.5.0 (Hermes Medical Solutions AB, Stockholm, Sweden). VOI delineation of the multiparametric images was performed by AHD using PMOD^®^ 4.0 (PMOD Technologies Ltd., Zürich, Switzerland). Semiquantitative values of SUV_max_ and SUV_mean_ were obtained from the conventional PET reconstructions, whereas MR_FDG_ and DV_FDG_ values were extracted from the multiparametric images.

A region of interest (VOI) was placed on the primary tumor and the FDG-avid lymph nodes. In patients with indications of cervical lymphadenopathy, the VOI was placed on the FDG-avid lymph nodes. In total, 142 VOIs on each of three images were analyzed.

**Figure 4:**
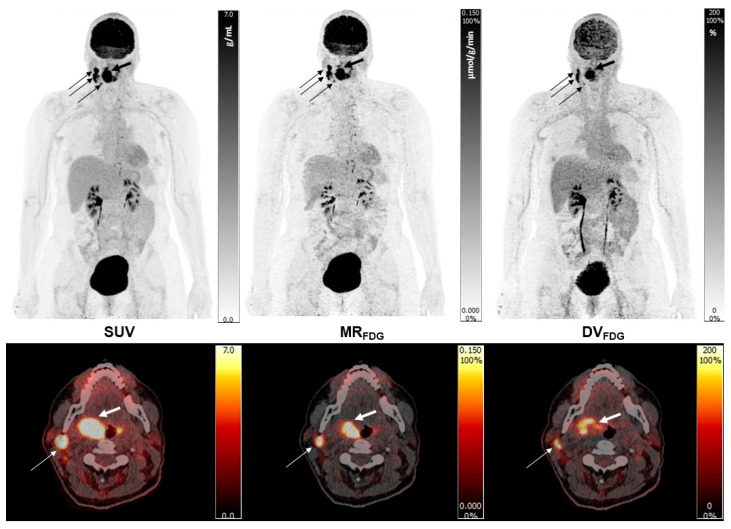
Example of SUV_bw_, MR_FDG_, and DV_FDG_ parametric images in a patient with right-sided oropharyngeal squamous cell carcinoma (SCC; thick arrows) and local lymph node metastases in neck levels II–IV (thin arrows). MR_FDG_ represents the irreversible uptake rate constant reflecting phosphorylated [^18^F]FDG-6-phosphate accumulation, while DV_FDG_ reflects the fractional volume of freely exchangeable, non-phosphorylated tracer and blood-pool activity. Together, these images demonstrate complementary kinetic information beyond static SUV_bw_ data.

Note the high quality of the Patlak images and the improved target to background activity of the MR_FDG_ images when compared to the SUV_bw_ images, as well as the marked activity in certain areas of the lesions on the DV_FDG_ images (Figure 4).

### 2.4. Statistical Analysis

Dynamic PET data from 43 patients were analyzed and categorized into two classes based on the presence or absence of malignancy. Average and maximum standardized uptake values ($SUV_{bw}$), as well as dynamic PET parameters (${MR}_{FDG}$ and ${DV}_{FDG}$), were measured in solid tumors or nodes.

A binomial logistic regression was performed to estimate the additional predictive value of dynamic PET in malignancy recognition. The optimal parameters $B_{C}=\left(\beta_{SUVC},\beta_{MRC},\beta_{DVC}\right)$ and $A_{C}$ of the logistic regression model, defined as

(1) $$P_{b}\left(C|M\right)=1-\frac{1}{1+e^{B_{C}M+A_{C}}}.$$

for predicting the probability of class C given a measured value M, were determined using the scikit-learn Python 3.13 module using lbfgs minimization [19]. Precision, recall (sensitivity), and F1-score, based on maximum probability decisions, were estimated for each class in the optimal model and for all carcinoma types combined.

In clinical applications, different probability thresholds could be used as decision criteria, considering subsequent treatment costs. To evaluate the model’s performance across this range of possible thresholds, the receiver operating characteristic (ROC) curve and the area under the curve (AUC) were determined for the optimal model. Probability thresholds for maximum Youden index and minimum distance to ideal point were found with corresponding sensitivity and specificity. Ninety-five percent confidence intervals (95% CIs) for diagnostic performance metrics (accuracy, sensitivity, and specificity) were calculated using the Wilson score method, based on binomial proportions. These intervals were computed for the main model (M1) and stratified subgroups (tumor and lymph node lesions) to better convey the robustness and precision of the estimates.

Six different models with various input data were studied:

(2) $$M_{1}=\left(SUV_{bw},{MR}_{FDG},{DV}_{FDG}\right),$$

(3) $$M_{2}=\left(SUV_{bw},{MR}_{FDG},{DV}_{FDG},SUV_{bw}\cdot {MR}_{FDG},SUV_{bw}\cdot {DV}_{FDG},{MR}_{FDG}\cdot {DV}_{FDG}\right),$$

(4) $$M_{3}=\left({MR}_{FDG},{DV}_{FDG}\right),$$

(5) $$M_{4}=\left(SUV_{bw}\right),$$

(6) $$M_{5}=\left({MR}_{FDG}\right),$$

(7) $$M_{6}=\left({DV}_{FDG}\right)$$

to estimate the additional predictive value of dynamic PET. The three parameters $SUV_{bw}$, ${MR}_{FDG}$, and ${DV}_{FDG}$ are expected to be linearly dependent under the assumptions underlying the Patlak plot. The models containing all three basic parameters may therefore be interpreted as a test of the condition’s validity: when the classification metrics show no additional benefit compared to use of only two of the parameters, linear dependency of the parameters can be assumed.

Formula (1) can be inverted for $B_{C}M$, so a threshold for *p*-value $T_{P}$ can be transformed to a threshold $T_{BM}$ for a simple linear expression in terms of $B_{C}M$:

(8) $$T_{BM}=\ln\left(\frac{1}{1-T_{P}}-1\right)-A_{C},$$

so that the conditions are equivalent.

(9) $$P_{b}\left(C|M\right)>T_{P}$$

(10) $$B_{C}M>T_{BM}$$

## 3. Results

Dynamic PET parameters were evaluated in a cohort of 43 patients, who were categorized into malignancy and non-malignancy lesion classes. Six binomial logistic regression models (M_1_–M_6_) were trained using combinations of average or maximum SUV_bw_, MR_FDG_ and DV_FDG_ to assess the predictive value of dynamic PET imaging.

**Figure 5:**
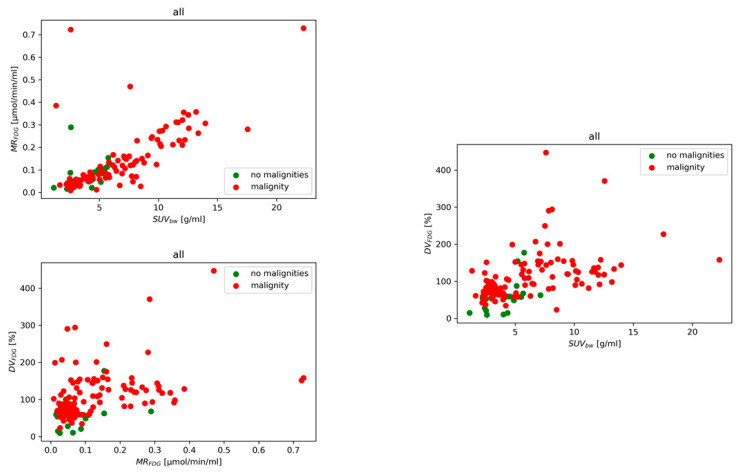
Lesion classification in parameter space using mean SUV_bw_, MR_FDG_, and DV_FDG_ values. MR_FDG_ quantifies tissue metabolic trapping of [^18^F]FDG (phosphorylated tracer fraction), whereas DV_FDG_ reflects the distribution volume of the reversible (non-phosphorylated) FDG fraction. The scatterplot illustrates the separation of malignant and benign lesions using multiparametric PET features.

Figure 5 shows the measured data in parameter space for patients categorized into the two classes. The optimal binomial regression model parameters $B_{C}$ and $A_{C}\;$ for six different models, M_1_–M_6_, are presented for the mean and maximum values (see Supplementary Tables S2 and S3). Performance metrics (precision, recall, and F1-score) for each mean value and each class are detailed in Supplementary Table S4. We also investigated the M_1_ analyzing node and tumor performance separately, with performance measures for comparison presented in Supplementary Table S5.

### 3.1. Diagnostic Performance of Models

Across all evaluated binomial logistic regression models, Model M_1_ (SUV_bw_, MR_FDG_, DV_FDG_) consistently showed the highest diagnostic performance in distinguishing malignant lesions from benign lesions. Using mean values, M1 achieved an accuracy of 0.82 (95% CI 0.75–0.89), sensitivity of 0.85 (95% CI 0.77–0.91), and specificity of 0.93 (95% CI 0.87–0.98), with a precision of 0.83, recall of 0.98, F1-score of 0.90, and specificity of 0.21. The models M_2_ and M_3_ demonstrated comparable performance with slightly lower or similar F1-scores and precision. Models based solely on dynamic parameters (M_3_, M_5_, and M_6_) provided similar classification performance to hybrid models, confirming the independent diagnostic value of kinetic features.

In the subgroup analysis (Supplementary Table S5), the classification performance further improved for solid tumors, with M_1_ reaching an F1-score of 0.92 and perfect recall (1.00). Performance in lymph nodes remained strong with an F1-score of 0.89. Models using maximum values showed slightly reduced but comparable diagnostic metrics.

### 3.2. ROC and Threshold Analysis

**Figure 6:**
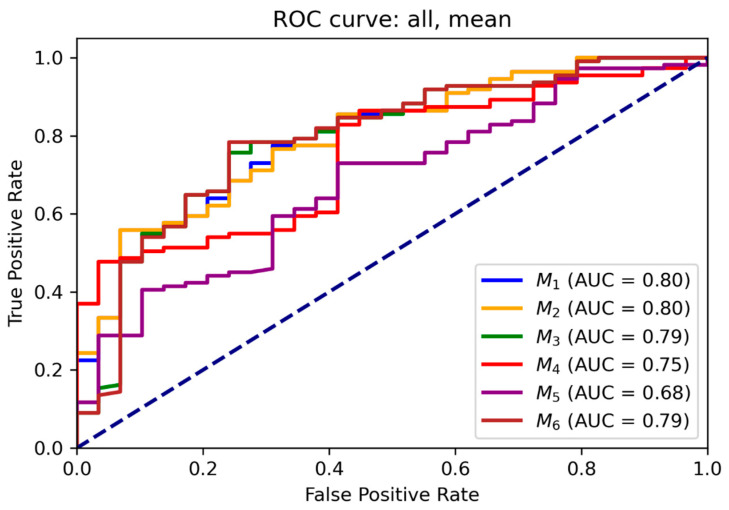
ROC curves for the binomial regression models for mean values. ROC analysis demonstrates diagnostic discrimination between malignant and benign lesions across combinations of SUV_bw_, MR_FDG_, and DV_FDG_.

**Figure 7:**
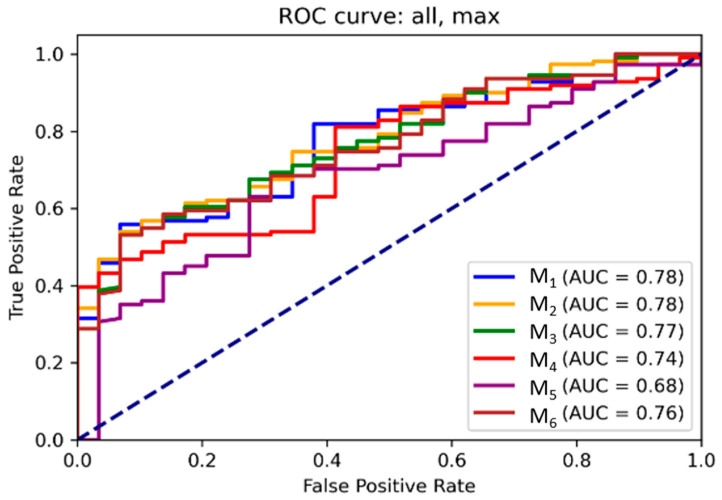
ROC curves for the binomial regression models for maximum values. As in Figure 5, MR_FDG_ represents the metabolic rate of [^18^F]FDG uptake (phosphorylated tracer fraction), while DV_FDG_ denotes the distribution volume of the non-phosphorylated tracer fraction. The curves illustrate the diagnostic performance of the six evaluated models (M1–M6) across varying probability thresholds.

Receiver operating characteristic (ROC) curves for each model are illustrated in Figure 6 and Figure 7 for mean and maximum values, respectively. Area under the ROC curve (AUC) values confirmed the superior performance of M_1_ across all input combinations.

Supplementary Tables S6 and S7 present optimal probability thresholds derived from the Youden index, minimum distance to the ideal point, and thresholds for achieving 95% sensitivity. For M_1_ using mean values, the maximum Youden index corresponded to a threshold of 0.85 (sensitivity: 0.56, specificity: 0.93), while the minimal distance threshold was 0.71. Comparable trends were observed for maximum values.

### 3.3. Relative Feature Contributions

For the relative contributions of each feature within the classification models, see Supplementary Table S8. In M_1_, DV_FDG_ accounted for 65% of the decision weight, SUV_bw_ for 34%, and MR_FDG_ for only 1%. In M_3_, DV_FDG_ contributed up to 97% of the model’s predictive value. These findings suggest that DV_FDG_ plays a prominent role in the predictive performance of the classification models.

### 3.4. Decision Thresholds and Clinical Translation

Linear threshold functions were derived to support clinical implementation. For Model M_3_, the optimal linear decision rule based on mean values was DV_FDG_ [%] + (7.2 × MR_FDG_ [µmol/min/mL]) > 70. For maximum values, the rule changed to DV_FDG_ [%] + (−29 × MR_FDG_ [µmol/min/mL]) > 194 (Table 2). Normalized single-parameter thresholds based on the Youden index for mean values were SUV_bw_ > 5.8 g/mL, MR_FDG_ > 0.050 µmol/mL/min, DV_FDG_ > 68% (Table 3).

## 4. Discussion

Our results clearly demonstrate that Patlak PET parameters, particularly MR_FDG_ and DV_FDG_, significantly improve the differentiation between malignant and non-malignant lesions compared to conventional SUV_bw_-based metrics. Among all evaluated models, the combination of mean SUV_bw_, MR_FDG_, and DV_FDG_ (Model M_1_) achieved the highest diagnostic performance, with an accuracy of 82% (95% CI 75–89%) and an F1-score of 0.90.

When stratified by lesion type, Model M1 performed better in solid tumors (accuracy 0.86 [95% CI 0.78–0.92]) than in lymph nodes (accuracy 0.81 [95% CI 0.73–0.88]), suggesting greater robustness in predicting malignancy in primary lesions compared to metastatic lymphadenopathy.

These findings are consistent with established knowledge that SUV_bw_ has limitations as a standalone quantitative biomarker in nuclear medicine. Its sensitivity to physiological and technical sources of variability, such as blood glucose levels, imaging time point, image noise, scanner resolution, and ROI delineation, can undermine reproducibility and diagnostic confidence [20]. In contrast, dynamic Patlak PET imaging allows for kinetic modeling of tracer uptake, offering a more biologically meaningful assessment of tissue metabolism, minimizing the impact of confounding factors like plasma glucose activity and imaging time-point dependence. Importantly, models using only dynamic parameter (Models M_3_, M_4_, and M_6_) performed comparably to hybrid models, underscoring the independent diagnostic utility of kinetic Patlak features.

Feature importance analysis (Supplementary Table S8) highlighted DV_FDG_ as the most prominent contributor in this model analysis. In Model M_1_, DV_FDG_ accounted for approximately 65% of the classification decision, compared to 34% for SUV_bw_ and only 1% for MR_FDG_. In Model M_3_, which included only MR_FDG_ and DV_FDG_, DV_FDG_ contributed to 97% of the decision weight. These findings are notable given that DV_FDG_ is an often overlooked parameter due to questions regarding its reproducibilty and clinical relevance [21], underscoring its potential diagnostic value in this context. Interestingly, more complex models (e.g., M_2_ and M_5_) that incorporated all three parameters did not outperform simpler two-parameter models. This may reflect underlying linear dependency among the features, as suggested by the Patlak graphical model, where over-parameterization could reduce interpretability without improving accuracy.

From a clinical perspective, these findings are particularly relevant for recurrent head and neck cancers, where anatomical distortion caused by surgery, reconstruction, radiation fibrosis, and inflammation complicate interpretation of conventional PET metrics. In such settings, improved lesion characterization using kinetic features can potentially reduce diagnostic uncertainty and help avoid unnecessary biopsies or surgical interventions. To support clinical translation, we proposed simplified linear decision thresholds based on DV_FDG_ and MR_FDG_ (Table 2) and suggested parameter cutoffs for malignancy detection using normalized data (Table 3) for mean values: SUV_bw_ > 5.8 g/mL, MR_FDG_ > 0.05 µmol/mL/min, DV_FDG_ > 68%. These values may serve as practical reference points, adaptable to desired tradeoffs between sensitivity and specificity in routine clinical use. These confidence intervals indicate that the observed diagnostic performance metrics are robust, with narrow uncertainty margins despite the relatively small sample size, supporting the reproducibility and potential clinical applicability of dynamic whole-body FDG PET/CT.

From a clinical standpoint, dynamic PET/CT parameters, such as DV_FDG_ metrics, can be meaningfully integrated into routine workflows to enhance diagnostic accuracy in key scenarios of head and neck oncology. In the evaluation of suspected recurrence, especially post-chemoradiotherapy, dynamic imaging can help distinguish viable tumor from post-treatment changes by capturing tracer kinetics rather than relying solely on static uptake values. In patients with CUP, dynamic parameters may improve lesion detectability or localization by identifying subtle metabolic activity not evident on conventional scans. Additionally, during treatment response assessment, dynamic imaging offers the potential for earlier detection of non-responders through quantitative analysis of metabolic flux, enabling earlier therapeutic adjustments. Since dynamic acquisitions are technically feasible with current PET/CT systems and require only modest protocol modifications, their incorporation into selected clinical workflows appears both practical and clinically valuable.

These considerations align with recent evidence highlighting the diagnostic limitations of conventional static FDG PET/CT in head and neck malignancies and the emerging potential of dynamic, multiparametric approaches to overcome them [22].

Compared to other malignancies, relatively few prior FDG PET studies in head and neck cancer have employed fully quantitative methodologies. This limitation is primarily due to the constraints of conventional PET/CT systems with standard axial field of view, which are suboptimal for acquiring dynamic data necessary for accurate input function estimation [23]. Advanced imaging protocols, such as continuous bed motion acquisition, are essential to overcome these challenges, as demonstrated in the authors’ previous studies [9]. It is pertinent to note that generating parametric images in head and neck cancer patients presents greater challenges compared to, for example, lung cancer patients. This disparity arises from the complex anatomy and motion artifacts in the head and neck region, which complicate the application of standard axial field-of-view scanners in this context. In contrast, lung cancer imaging benefits from the use of total-body PET scanners, which provide high temporal resolution and enable dual-blood input function modeling, facilitating more accurate kinetic analysis and parametric imaging [24].

Previous research on dynamic PET imaging in head and neck SCC has explored various approaches to improve lesion characterization and treatment planning. Early dual time-point imaging studies aimed to enhance discrimination between benign and malignant lesions but achieved only modest diagnostic improvements [25,26,27]. Subsequent pilot studies applied dynamic PET/CT for theoretical radiotherapy volume delineation in oropharyngeal cancer, demonstrating the potential of kinetic parameters to capture metabolic heterogeneity beyond static SUV_bw_ measures [25,26,27].

More recently, integration of molecular biomarkers with functional imaging has emerged as a promising direction. A significant positive correlation between circulating tumor DNA (ctDNA) and total lesion glycolysis (TLG) derived from FDG PET/CT was reported in HNSCC patients, suggesting that combined molecular and imaging biomarkers can enhance prognostic accuracy and disease monitoring [28].

Our study advances this evolving field by employing a D-WB PET/CT protocol with the multiparametric Patlak-derived parameters MR_FDG_ and DV_FDG_, combined with conventional SUV_bw_, to characterize both tumors and nodal metastases. The high diagnostic accuracy (82% [95% CI 0.75–0.89]) and specificity (93% [95% CI 0.87–0.98]) observed, along with the prominent contribution of DV_FDG_, underscore the clinical potential of this approach in differentiating malignant lesions from benign lesions. These findings highlight the complementary nature of kinetic and molecular imaging biomarkers and support further research integrating these modalities to improve diagnostic precision and personalized treatment strategies in HNSCC.

This study has several limitations. First, the small sample size (*n* = 43) restricts the generalizability of the findings. Second, lesions were not stratified by histological subtype, anatomical subsite, or other factors that may influence tracer kinetics. Third, although kinetic parameters were derived using the Patlak model, which assumes irreversible tracer uptake over time, a behavior generally observed in malignant lesions, we did not independently validate this assumption. In lesions with necrosis, disrupted vasculature, or heterogeneous perfusion, the Patlak model’s assumptions, particularly the irreversibility of tracer uptake, may not be satisfied. This could lead to inaccurate MR_FDG_ and DV_FDG_ estimates, as potential reversible tracer or nonlinear kinetics are not accounted for in the model. Additionally, we did not compare Patlak modeling with alternative kinetic approaches, such as full compartmental models (LAFOV PET) or nonlinear regression methods, which might better capture complex tracer dynamics in selected lesions. Another limitation of the study is the inclusion of a heterogeneous cohort comprising different histological tumor types as well as both staging and post-therapeutic scans. However, this approach was intentional in order to reflect the diversity encountered in real-world clinical practice and to demonstrate the robustness of the method across a wide spectrum of clinical scenarios. Lastly, a limitation of our study is the lack of available data on HPV status for patients with oropharyngeal cancers. Since HPV positivity is known to influence the metabolic behavior of these tumors, the absence of this information may affect the interpretation of our findings. Future studies should incorporate HPV status to better understand its impact on dynamic [^18^F]FDG-PET/CT parameters and improve diagnostic accuracy [29].

Future research should aim to validate these results in larger, multicenter cohorts, with stratification by tumor subtype, treatment history, and anatomical context. Given the specific assumptions of the Patlak model, further studies should evaluate its applicability across different lesion types and explore alternative or hybrid kinetic modeling approaches that may better capture complex tracer dynamics. Moreover, integration of dynamic PET data with multimodal imaging, radiomics, or molecular biomarkers could enhance diagnostic accuracy in complex post-treatment settings. Development of clinical decision-support tools based on dynamic metrics may aid tumor boards in real-time decision-making. Assessing automation feasibility, model reproducibility, and clinician acceptance will be critical for translation into practice. Ultimately, prospective trials are needed to determine whether incorporating dynamic PET metrics into clinical workflows improves key outcomes including diagnostic accuracy, treatment decisions, avoidance of invasive procedures, patient quality of life, and healthcare cost-effectiveness.

Finally, the cost-effectiveness and feasibility of implementing dynamic whole-body PET/CT protocols in routine clinical practice remain to be fully evaluated. Dynamic imaging requires longer acquisition times, dedicated reconstruction algorithms, and additional data processing, all of which may limit its widespread adoption compared with conventional static PET/CT. However, as scanner technology advances and fully automated reconstruction pipelines become more accessible, the integration of dynamic protocols may become increasingly practical and economically justified in select oncologic settings.

## 5. Conclusions

Dynamic PET data appears to enhance the distinction between malignant and non-malignant lesions, particularly in solid tumors. This study provides evidence for the diagnostic value of dynamic imaging parameters in classifying malignant lesions. Logistic regression models that incorporate a limited number of kinetic features demonstrate a favorable balance between accuracy and interpretability, highlighting their potential as decision-support tools in clinical practice. Moreover, the identification of clinically relevant thresholds may enable more reliable detection of tumor recurrence. Future studies with larger cohorts are warranted to further validate these findings and confirm their clinical applicability.

## Figures and Tables

**Table 1:** Histology of examined lesions.

Histology	Tumor (*n* = 38)	Lymph Nodes (*n* = 104)
Malignant	30 (78.9%)	82 (78.8%)
Squamous cell carcinoma	25 (65.8%)	62 (50.6%)
Other malignities (lymphoma, adenocarcinoma, verucosic carcinoma, epithelial-myoepithelial carcinoma, epitheloid sarcoma, sebocellular carcinoma)	5 (13.2%)	20 (19.2%)
Non-malignant (e.g., inflammation, physiological finding)	8 (21%)	22 (21.1%)

**Table 2:** Thresholds derived from maximum Youden index, minimum distance to ideal point and for 95% sensitivity for dynamic PET. The thresholds are derived for the following equation: DV_FDG_ [%] + weight × ${MR}_{FDG}$ [µmol/min/mL] > threshold.

M_3_	Weight for MR_FDG_	Youden Index	Distance	95% Sensitivity
mean	7.2	70	70	52
max	−29	194	147	94

**Table 3:** Normalized thresholds $T_{M}=T_{B}M/B$ derived from maximum Youden index and minimum distance to ideal point and for 95% sensitivity for individual parameters.

	Unit	Youden Index	Distance	95.0% Sensitivity
mean	SUV_bw_ (g/mL)	5.8	3.0	2.4
	MR_FDG_ (µmol/mL/min)	0.050	0.050	0.026
	DV_FDG_ (%)	68	68	51
max	SUV_bw_ (g/mL)	8.7	4.4	3.4
	MR_FDG_ (µmol/mL/min)	0.110	0.110	0.051
	DV_FDG_ (%)	202	168	96

The normalized thresholds presented above were derived from the Youden index using mean parameter values and indicate optimal cut-offs for distinguishing malignant lesions from benign lesions. These thresholds may serve as practical guidance in clinical [^18^F]FDG PET/CT interpretation, aiding differentiation between malignant and non-malignant tissue in head and neck oncology. However, further validation in larger cohorts is necessary before clinical implementation.

## Data Availability

The datasets used and analyzed during the current study are available from the corresponding author on reasonable request.

## Supplementary Materials

The following supporting information can be downloaded at https://www.mdpi.com/article/10.3390/diagnostics15202651/s1: Supplementary Table S1: Clinical Indications for Imaging/Diagnostic Evaluation; Table S2: Optimal model parameters as a result of binomial logistic regression of all patients using mean value; Table S3: Optimal model parameters as a result of binomial logistic regression of all patients using maximum value; Table S4: Overall accuracy, precision, sensitivity, f1-score and specificity for maximum probability decision for the binomial regression models M1–M6 using mean value; Table S5: Overall accuracy, precision, sensitivity, f1-score and specificity for maximum probability decision for the binomial regression model M1 for mean and maximum values measured in solid tumors only, nodes only and all data combined; Table S6: Probability thresholds for maximum Youden index, minimum distance to ideal point and for 95% sensitivity and corresponding specificity and sensitivity for the binomial regression models using mean value; Table S7: Probability thresholds for maximum Youden index, minimum distance to ideal point and for 95% sensitivity and corresponding specificity and sensitivity for the binomial regression models using maximum value; Table S8: Relative contribution of the PET parameters to classification within the models.
